# Fabrication of ZnCoO nanowires and characterization of their magnetic properties

**DOI:** 10.1186/1556-276X-9-221

**Published:** 2014-05-07

**Authors:** Bum-Su Kim, Seunghun Lee, Won-Kyung Kim, Ji-Hun Park, Yong Chan Cho, Jungdae Kim, Chae Ryong Cho, Se-Young Jeong

**Affiliations:** 1 Department of Cogno-Mechatronics Engineering, Pusan National University, 1268-50, Samnangin-ro, Samnangjin-eup, Miryang 627-706, Republic of Korea; 2 The Institute of Basic Science, Korea University, Seoul 136-713, Republic of Korea; 3 Crystal Bank Institute, Pusan National University, 1268-50, Samnangin-ro, Samnangjin-eup, Miryang 627-706, Republic of Korea; 4 Department of Physics, University of Ulsan, 93 Daehak-ro, Nam-gu, Ulsan 680-749, Republic of Korea; 5 Department of Nano Fusion Technology, Pusan National University, Samnangin-ro 1268-50, Republic of Korea

**Keywords:** ZnCoO, Nanowire, Solution aqueous method, Ferromagnetism

## Abstract

Hydrogen-treated ZnCoO shows magnetic behavior, which is related to the formation of Co-H-Co complexes. However, it is not well known how the complexes are connected to each other and with what directional behavior they are ordered. In this point of view, ZnCoO nanowire is an ideal system for the study of the magnetic anisotropy. ZnCoO nanowire was fabricated by trioctylamine solution method under different ambient gases. We found that the oxidation of trioctylamine plays an essential role on the synthesis of high-quality ZnCoO nanowires. The hydrogen injection to ZnCoO nanowires induced ferromagnetism with larger magnetization than ZnCoO powders, while becoming paramagnetic after vacuum heat treatment. Strong ferromagnetism of nanowires can be explained by the percolation of Co-H-Co complexes along the *c*-axis.

## Background

Co-doped ZnO (ZnCoO) has been intensively studied because of its widespread applicability as a magnetic semiconductor [1-3]. Many studies have shown that its ferromagnetism depends on the fabrication method and the post-treatment conditions. A variety of theoretical models have been suggested to explain experimental results [2,4-7]. However, the origin of ZnCoO ferromagnetism remains unclear.

Chemical fabrication of ZnCoO is greatly affected by experimental factors, compared with other deposition methods such as pulsed laser deposition and radio frequency (RF) sputtering [8-11]. Post heat treatment, used to eliminate organic residuals, can induce secondary phases and crystalline defects, which can interfere with the investigation of intrinsic properties [12-15]. Unwanted hydrogen contamination during fabrication, in particular, is known to create defects that degrade the physical properties of ZnO-based materials. However, many experimental results have consistently supported the model of magnetic semiconductors in which Co-H-Co complexes are created by hydrogen doping of ZnCoO [5,13,16-21].

ZnCoO nanowires have received extensive attention because of advantages such as high aspect ratio and widespread applicability [22-25]. However, determining the intrinsic properties has been difficult, and the performance and reliability of ZnCoO nanowire devices have been controversial because they are typically fabricated using chemical methods with non-polar solvents [23,26].

ZnCoO nanowire fabrication with non-polar solvents is based on thermal decomposition via a well-known chemical mechanism [27-30]. The reported fabrication conditions, including temperature, additives, and reaction environment, vary [26,31]. These factors affect not only the growth of the nanowires but also the physical properties of the final nanowires. Although ambient synthesis has been regarded as a significant condition in such chemical reactions [32], no one has yet reported on the properties of nanowires with respect to their synthesis environment. In this study, we examined the change in the nanowire morphology as a function of the fabrication conditions. This is the first report suggesting that the ambient gas should be carefully considered as one of the more important factors in the chemical synthesis of high-quality nanowires. The high-quality ZnCoO nanowires initially exhibited intrinsic paramagnetic behavior; however, following hydrogen injection, the nanowires became ferromagnetic. This finding is consistent with the hydrogen-mediation model. Additionally, this was the first observation of the superb ferromagnetism of the nanowire, compared with powders, reflecting the favored direction of the ferromagnetism along the *c*-axis of the nanowires.

## Methods

**Figure 1:**
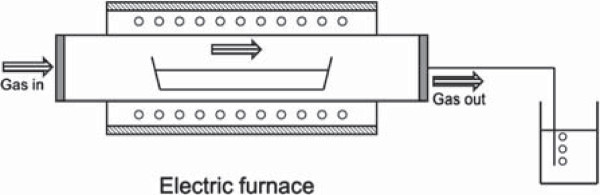
Electric furnace for the synthesis of ZnCoO nanowires.

For the fabrication of Zn_0.9_*Co*_0.1_O nanowires in this study, we chose the aqueous solution method, which is one of the representative chemical fabrication routes. Zinc acetate (Zn(CH_3_CO_2_)_2_) (2.43 mmol) and cobalt acetate (Co(CH_3_CO_2_)_2_) (0.27 mmol) were used as precursors, and non-polar trioctylamine (N(CH_3_(CH_2_)_7_)_3_) (25 ml) was used as the solvent; Co doping of ZnO was accomplished using 10 mol.% cobalt acetate. The precursors were rapidly heated to 310°C in an electric furnace with an inert gas atmosphere for fast thermal decomposition (Figure 1). The syntheses were carried out using different ambient gases, including flowing inert Ar (99.999%), flowing air (99.999%) with a continuous oxygen supply, and closed air (99.999%) with oxygen inclusion only for the initial reaction (Table 1). The gas flow rate was maintained at 25 sccm. The nanowire length was manipulated from 500 nm to 3 μm by controlling the synthesis time between 30 min and 2 h. The synthesized nanowires were cleaned in ethanol and distilled water repeatedly, followed by annealing in stages at 300°C for 10 h and 800°C for 10 h under a vacuum (10^-2^ Torr) to remove organic residues. For comparison, ZnCoO nanopowder [13] and ZnCoO micropowder [20] were also prepared (see the references for detailed information). Hydrogen injection was performed by plasma treatment using an Ar/H (8:2) mixed gas (99.999%), and all samples were exposed twice for 15 min to hydrogen plasma using an RF power of 80 W.

**Table 1:** Controlling ambient gas by gas distinction

**Sample name**	**Gas**
S1	Argon gas (99.999%, continuous flow)
S2	Air gas (99.999%, continuous flow)
S3	Air gas (99.999%, non-continuous)

The change in nanowire morphology and the secondary phase were investigated by field-emission scanning electron microscopy (FE-SEM, S-4700, Hitachi, Tokyo, Japan) and X-ray diffraction (XRD, Empyrean series2, PANalytical, Almelo, The Netherlands). Magnetic properties such as magnetization were measured using a vibrating sample magnetometer (VSM, model 6000, Quantum Design, San Diego, CA, USA) attached to a physical property measurement system.

## Results and discussion

**Figure 2:**
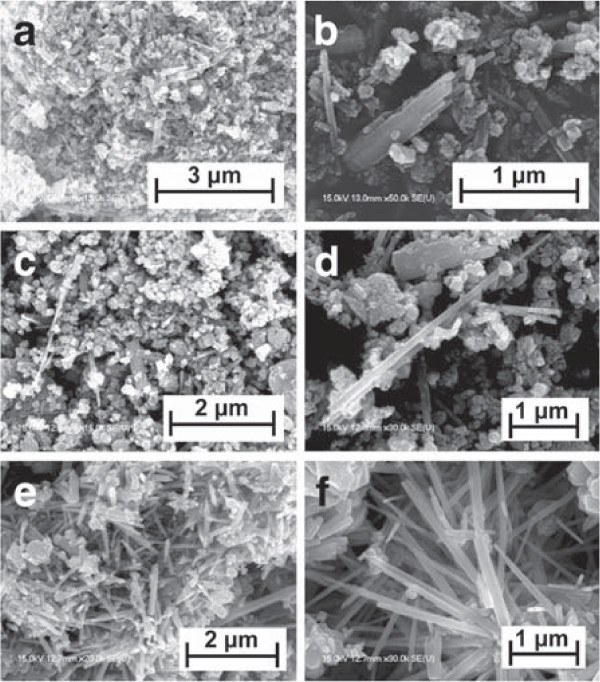
**FE-SEM images of ZnCoO nanowires fabricated using different ambient gases.** **(a)** FE-SEM image of sample S1 obtained under continuous argon gas flow and **(b)** a magnified image. **(c)** FE-SEM image of sample S2 obtained under continuous air gas flow including oxygen and **(d)** a magnified image. **(e)** FE-SEM image of sample S3 obtained under initial air gas conditions without continuous air gas flow and **(f)** a magnified image.

Figure 2 shows the FE-SEM images of the ZnCoO nanowires synthesized using different ambient gases. Figure 2a shows the FE-SEM images of the samples labeled S1, which were fabricated using ambient Ar gas. Figure 2b shows the same image magnified by a factor of three. ZnCoO nanowires were produced sporadically, and the average length was 700 nm. Figure 2c shows the FE-SEM images of the samples labeled S2, which were fabricated using air continuously supplied with oxygen. Figure 2d shows the same image magnified by a factor of three. ZnCoO nanowires were produced sporadically, and the maximum length was approximately 2.5 μm. Figure 2e shows the FE-SEM images of the samples labeled S3, which were generated using a fixed air supply with restricted oxygen content. Figure 2f shows the same image magnified by 1.5. The ZnCoO nanowires were produced uniformly, and the average length was 2 μm. These results indicate that the morphology of the ZnCoO nanowires depends on the ambient gas and, in particular, on the oxygen content.

**Figure 3:**
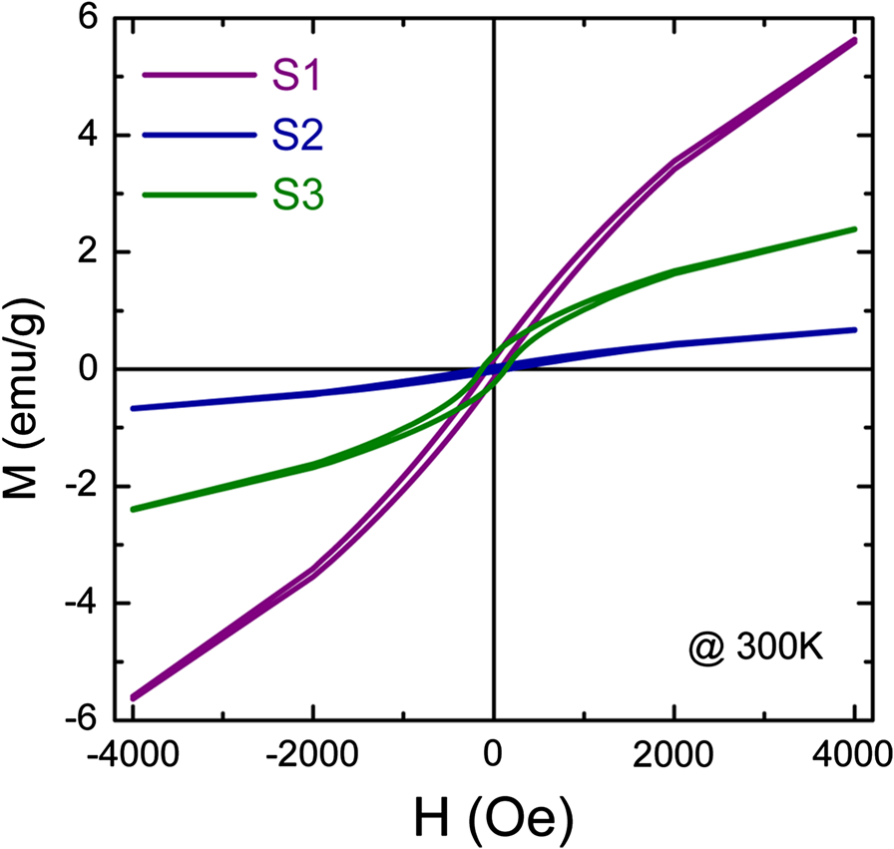
***M-H* curves of the as-grown ZnCoO nanowires.** *M-H* characteristics of ZnCoO nanowires fabricated using different ambient gases. The *M-H* curves were acquired at 300 K.

XRD confirmed that the fabricated samples (S1, S2, and S3) contained no Co-related species and that all peaks corresponded to a single ZnO phase. Figure 3 shows magnetization-applied magnetic field (*M*-*H*) curves measured by the VSM at room temperature. Different ferromagnetic hysteresis shapes were observed for the three samples, even though they contained equal amounts of Co. This means that the ferromagnetism of ZnCoO nanowires is closely related to the synthesis environment. Therefore, we investigated the dependence of the ferromagnetism on the ambient gas during ZnCoO nanowire fabrication.

Oxidation of trioctylamine solution was considered as a possible explanation for the different morphologies and properties of ZnCoO nanowires depending on ambient gases. It was expected that trioctylamine would react with oxygen at 310°C, near the boiling point, and then trioctylamine oxide would be formed via the following reaction:

(1) $$2{\left(C_{2}H_{5}\right)}_{3}N(g)+O_{2}(g)\to 2{\left(C_{2}H_{5}\right)}_{3}N^{+}-O^{-}$$

**Figure 4:**
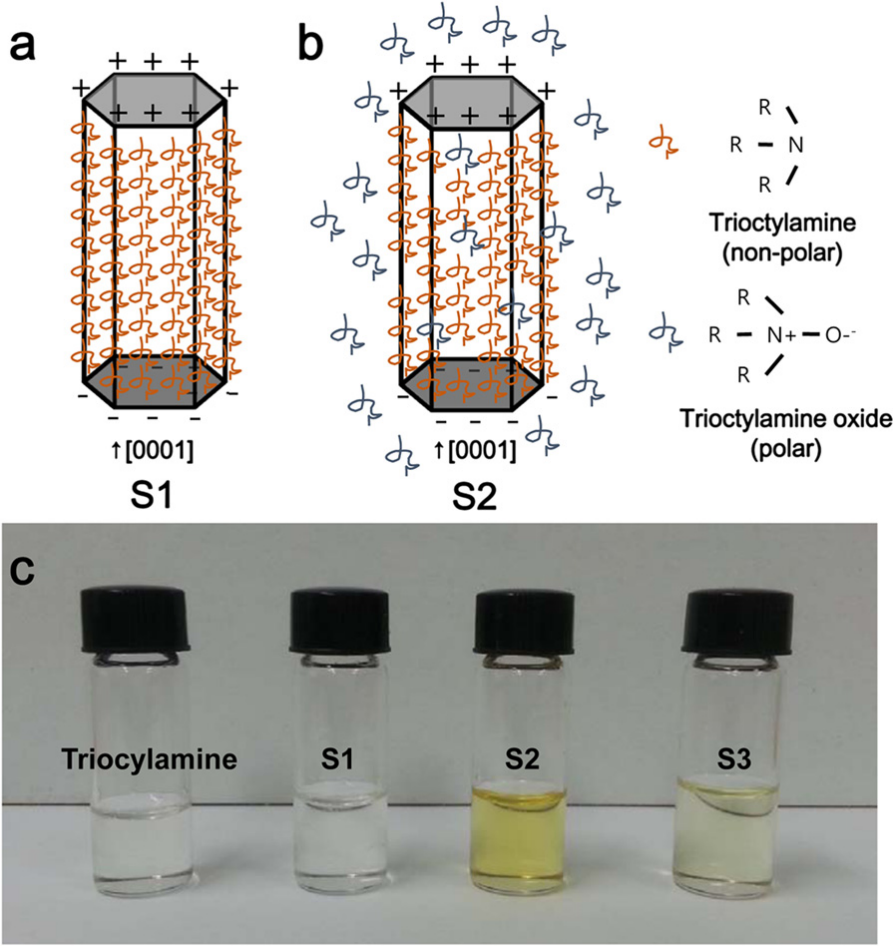
**Schematics illustrating the growth processes of ZnCoO nanowires and photographs of trioctylamine solution.** Under **(a)** Ar and **(b)** air ambient gas. Oxidation of trioctylamine in (b) produces polar amine oxides. **(c)** Photographs of trioctylamine solution and the ZnCoO nanowire solutions, showing the different colors during the reaction, depending on O_2_ content.

The amine oxides generated by the oxidation reaction are polar, allowing them to act as surfactants [33]. The (0001) planes of ZnCoO have relatively low surface energy because of the dangling bonds that induce surface polarity, as shown in Figure 4a. The trioctylamine non-polar solution provides a favorable environment for the growth of nanowires along the *c*-axis, because the plane parallel to the *c*-axis of ZnCoO has lower surface energy and a different polarity compared with the perpendicular plane [34,35]. In the case of S2, the oxidation reaction occurred continuously, and the amine oxides were generated in excess, as shown in Figure 4b. The excessive formation of amine oxides could change the polarity of the solution from non-polar to polar and hinder the growth of the *c*-axis-oriented ZnCoO nanowires. However, the correct amount of amine oxides generated in sample S3, in which oxygen gas was supplied only initially, positively affected the synthesis of ZnCoO nanowires. In many studies, oleic acid, a well-known surfactant, was intentionally added during the fabrication of ZnCoO nanowires [36]. In our study, the growth of nanowires was enhanced simply by controlling the ambient gas instead of supplying additional surfactant.

Figure 4c shows color changes during the reaction, as the solution turned brown after the synthesis of nanowires under each ambient gas. Generally, such browning reaction results from the oxidation of the chemical specimen. Because the color brightness is dependent on the oxygen content during the synthesis reaction, we assumed that the browning originated from the creation of the oxidized specimen in the presence of trioctylamine. The formation of an amine oxide specimen can be a contributing factor in the determination of the ZnCoO nanowire morphology. Therefore, we suppose that the variation in the synthesized ZnCoO nanowires shown in Figure 2 is the result of different amine oxide contents generated under different ambient gases.

**Figure 5:**
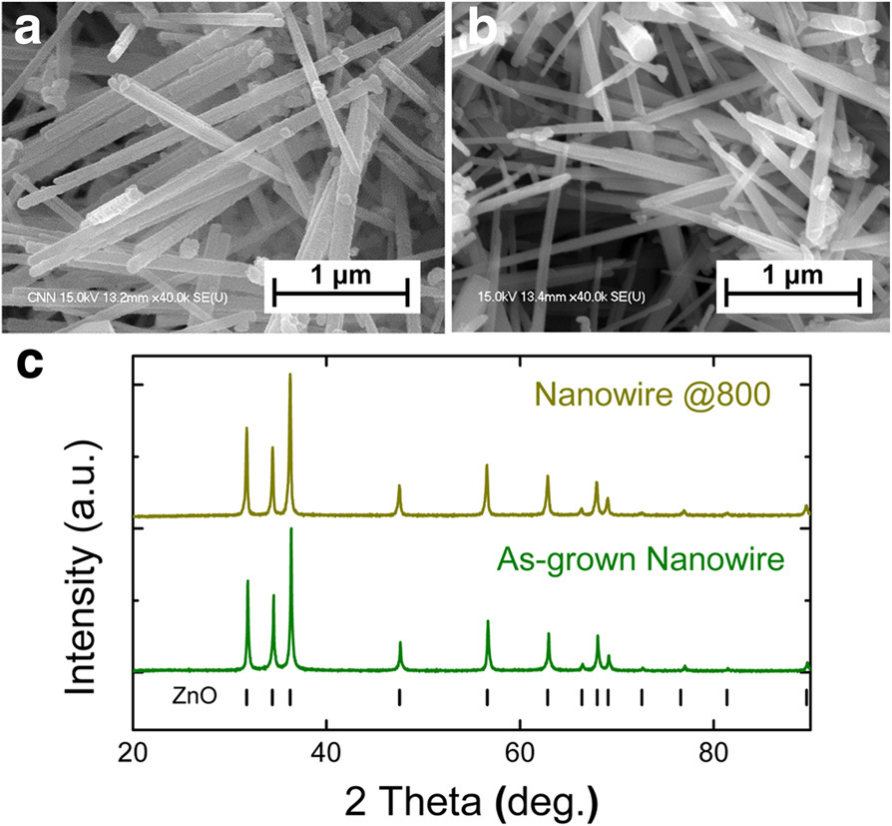
**FE-SEM image and XRD patterns of ZnCoO nanowire.** FE-SEM image of ZnCoO nanowire **(a)** before annealing (As-grown Nanowire) and **(b)** after vacuum annealing process at 800°C (Nanowire at @800). **(c)** XRD patterns of ZnCoO nanowire before and after the thermal treatment.

It has been reported that ZnCoO doves not exhibit intrinsic ferromagnetism, whereas our as-grown nanowires showed clear ferromagnetic hysteresis, as shown in Figure 3. For more detailed analysis of the intrinsic properties of ZnCoO nanowires, vacuum annealing was performed at 800°C on S3 ZnCoO nanowires. Figure 5a,b shows the FE-SEM images of the ZnCoO nanowires as grown and after the annealing treatment. The nanowires retained their shape after heat treatment at 800°C, with no noticeable change in morphology. Figure 5c shows the XRD patterns of ZnCoO nanowires as grown and after annealing. All patterns correspond to those of a single ZnO phase, and no secondary phases were observed within the detection limit. The full-width at half maximum values of the peaks did not change after annealing, indicating that the size of the nanowires did not change significantly after the heat treatment.

**Figure 6:**
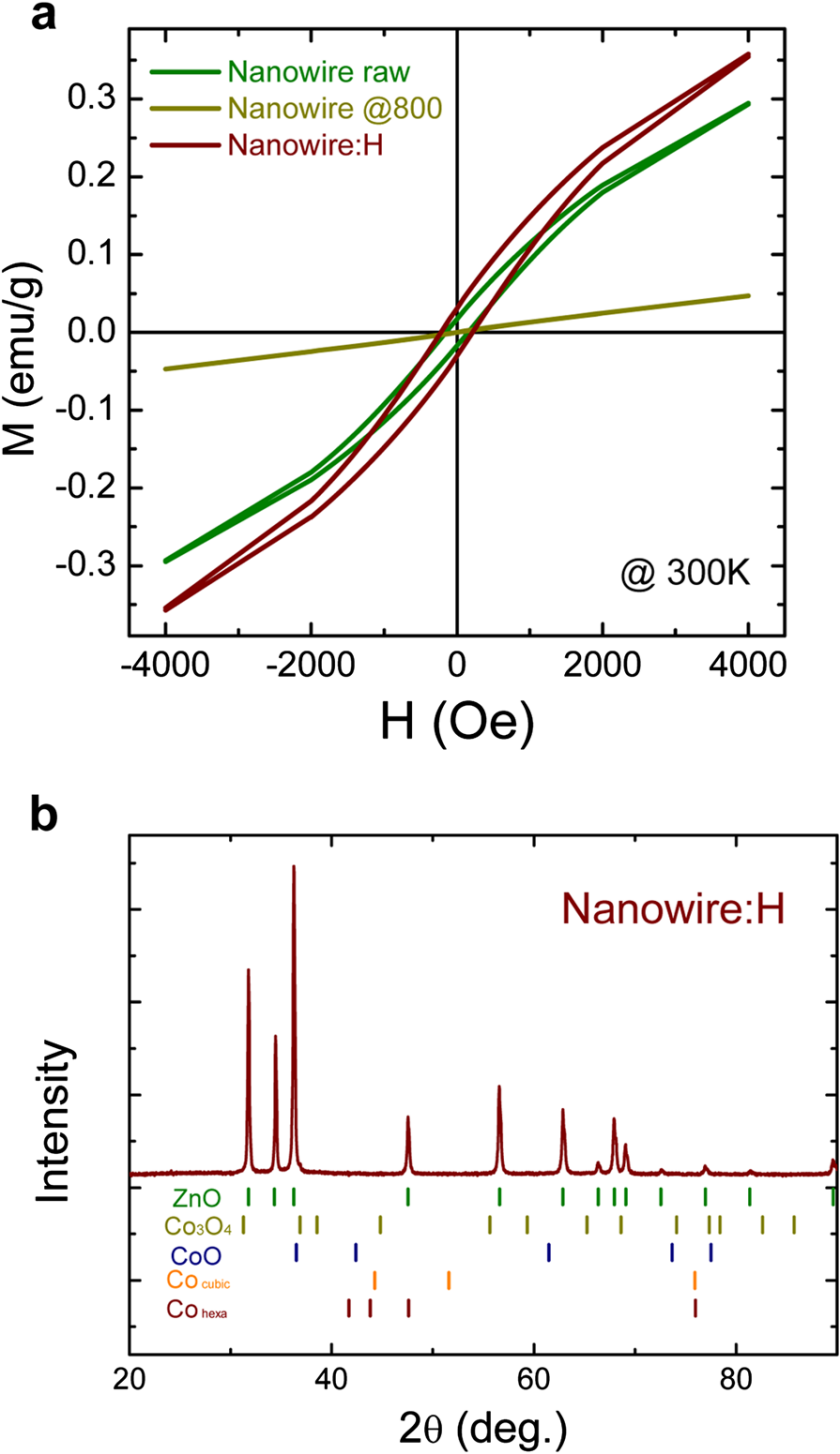
***M-H* curves and XRD patterns of ZnCoO nanowire.** **(a)** *M-H* curves of the as-grown nanowire without annealing (Nanowire raw), nanowire after vacuum annealing at 800°C (Nanowire @800), and nanowire after hydrogen treatment of the vacuum-annealed nanowire at 800°C (Nanowire:H), respectively. **(b)** XRD patterns of hydrogenated ZnCoO nanowire (Nanowire:H).

Figure 6a shows the *M-H* curves of the ZnCoO nanowires before and after heat treatment and subsequent hydrogen plasma treatment. Before heat treatment, the nanowires showed a clear ferromagnetic hysteresis, but the curves became completely paramagnetic after heat treatment at 800°C. We assumed that the ferromagnetic behavior observed in the nanowires before thermal heat treatment was attributed to (Co related-) organic residue on the surface of the nanowires synthesized via the aqueous solution method [15,20,37]. However, a more detailed analysis of the surface composition would require an additional investigation utilizing a surface characterization technique, such as XPS or Raman spectroscopy. It was evident that the vacuum heat treatment effectively eliminated the (Co related-) organic residue, and the pure ZnCoO nanowires without (Co related-) organic residue exhibited paramagnetic properties [20,38,39]. The paramagnetic behavior became ferromagnetic after hydrogen plasma treatment. The ferromagnetic hysteresis curve itself was similar to those of the as-grown nanowires, but the origin of the ferromagnetism was different. This result is also consistent with previous studies suggesting that hydrogen mediates ferromagnetism in ZnCoO by the formation of a C-H-Co complex. Figure 6b shows an XRD pattern of nanowires after hydrogen treatment, where all the diffraction peaks correspond to those of a single ZnO phase with no Co secondary phases. Considering the above results, the ferromagnetism of ZnCoO nanowires grown by Yuhas et al. [26] using the same aqueous solution method was attributed to surface contamination by hydrogen compounds, such as organic residue. Therefore, it should be noted that the magnetic characteristics of the as-grown ZnCoO nanowires fabricated using the aqueous solution method are not intrinsic but are due to surface contamination.

**Figure 7:**
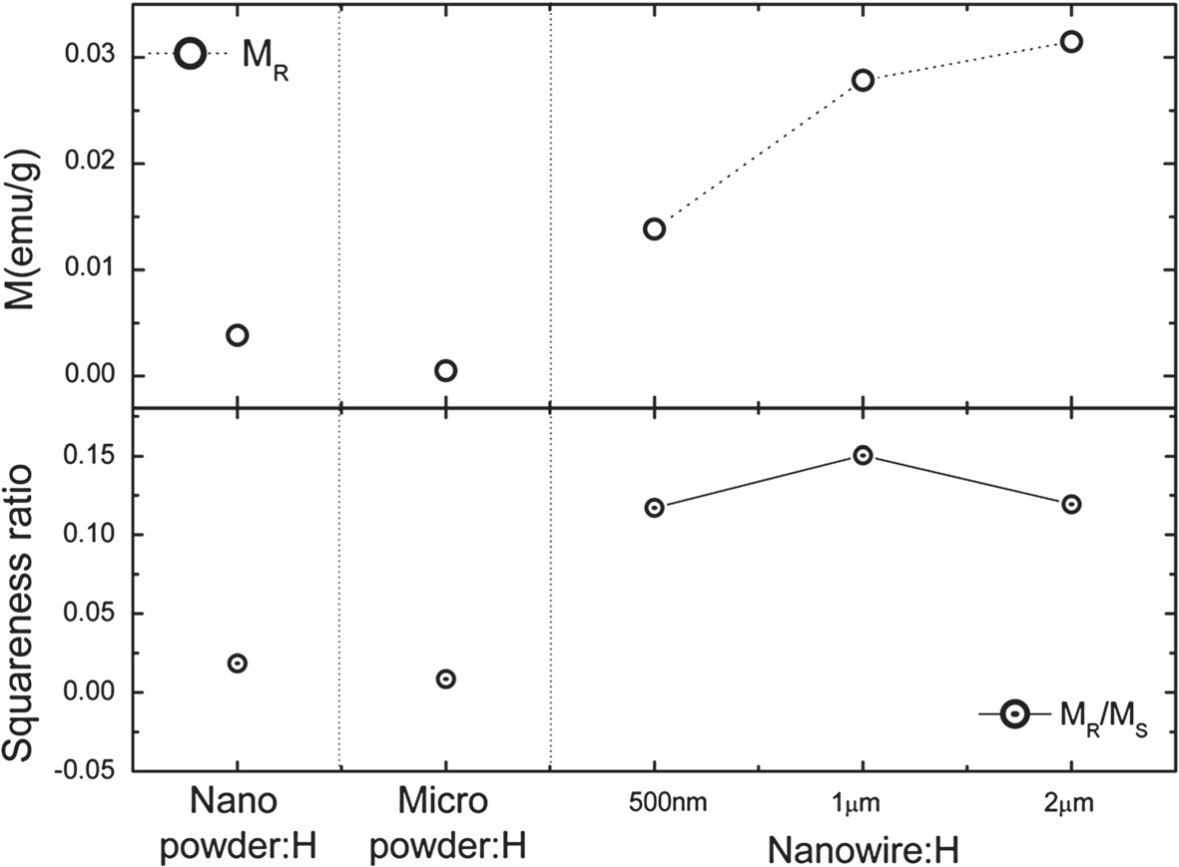
**Magnetic properties depending on the different shapes and sizes of ZnCoO:H.** Each ZnCoO hydrogenated at 80 W (Nanopowder:H, Micropowder:H, and Nanowire:H). Nanowire:H shows relatively higher *M*_R_ and squareness ratio (*M*_R_/*M*_S_) than Nanopowder:H and Micropowder:H.

To determine the direction of the spin ordering, we compared the ferromagnetic *M-H* curves of the nanowires, nanopowder, and micropowder for 10 mol% Co-doped ZnO under the same hydrogen injection conditions. The nano- and micro-powder samples had diameters of 20 nm and 1 μm, respectively. The lengths of the nanowires were manipulated from 0.5 to 2 μm, while the diameter was constant at 40 nm, by varying the synthesis processing time. Figure 7 shows the magnetic characteristics of the samples obtained from VSM measurements. The *c*-axis-oriented nanowires showed increasing magnetization with increasing nanowire length, as well as the largest remnant magnetization (*M*_R_) compared to the powder samples. The ZnCoO nanowires showed a higher squareness ratio (*M*_R_/*M*_S_) (more than 10 times compared with the other samples). It has been reported that squareness ratio is related to the magnetic domain size formed by the ferromagnetic units [13,15,40]. In previous studies, ferromagnetic models suggested that hydrogen was introduced by Co-H-Co complexes [5], but these reports did not fully explain how the complexes were ordered and aligned. We found that the ferromagnetism in nanowires depended on the nanowire length and was greatly enhanced compared to that of nano- and micro-powders. Such results imply that magnetic ordering in ZnCoO nanowires occurs preferentially along the *c*-axis due to the percolation of the Co-H-Co complex unit.

## Conclusions

High-quality ZnCoO nanowires were obtained by the aqueous solution method. The ambient gas affected the magnetic properties of the fabricated samples, and the oxidation of trioctylamine solution played an important role. The generation of an appropriate amount of amine oxide due to a limited oxygen supply enhanced the growth of ZnCoO nanowires because the amine oxide acted as a surfactant. However, excessive oxygen inhibited the growth by changing the polarity of the solution. The as-grown ZnCoO nanowires exhibited magnetic properties, but these properties were extrinsic due to the thermal heat treatment process. Intrinsic ferromagnetism in ZnCoO nanowires was only obtained after hydrogen treatment. The room-temperature ferromagnetism of nanowires grown along the *c*-axis was larger than those of the nano- and micro-powders.

We suggest that the magnetic units of Co-H-Co formed in ZnCoO percolated efficiently along the *c*-axis. Furthermore, we expect that the nanowire structure of ZnCoO will enable further studies of magnetic anisotropy.

## Competing interests

The authors declare that they have no competing interests.

## Authors’ contributions

BSK and SL designed and planned the experiments. BSK performed powder and nanowire synthesis and measurements. BSK, SL, and SYJ performed data analysis and interpretation. WKK, JHP, and YCC assisted with sample characterization and contributed to measurement discussions. JK, CRC, and SYJ wrote the manuscript with help from the co-authors. All authors discussed the results and reviewed the manuscript. All authors read and approved the final manuscript.

## Authors’ information

BSK, WKK, and JHP are graduate students of the Department of Cogno-Mechatronics Engineering, Pusan National University, Republic of Korea. SL is a research professor at the Institute of Basic Science, Korea University, Republic of Korea. YCC is a research professor at the Crystal Bank Institute, Pusan National University, Republic of Korea. JK is an associate professor at the Department of Physics, University of Ulsan, Republic of Korea. CRC is an associate professor at the Department of Nano Fusion Technology, Pusan National University, Republic of Korea. SYJ, the corresponding author, is a professor at the Department of Cogno-Mechatronics Engineering, Pusan National University, Republic of Korea.
